# A Model for Empowering Rural Solutions for Cervical Cancer Prevention (He Tapu Te Whare Tangata): Protocol for a Cluster Randomized Crossover Trial

**DOI:** 10.2196/51643

**Published:** 2023-09-14

**Authors:** Beverley Lawton, Evelyn Jane MacDonald, Francesca Storey, Jo-Ann Stanton, Anna Adcock, Melanie Gibson, Varsha Parag, Ngaire Kereru Sparkes, Bobby Kaimoana, Frances King, Marion Terry, Huti Watson, Matthew Bennett, Charles Seymour Lambert, Stacie Geller, Isitokia Paasi, Merilyn Hibma, Peter Sykes, David Hawkes, Marion Saville

**Affiliations:** 1 National Women's Health Research Centre Faculty of Health Victoria University of Wellington Wellington New Zealand; 2 National Institute for Health Innovation School of Population Health University of Auckland Auckland New Zealand; 3 Queen Street Practice Wairoa New Zealand; 4 Ngāti Porou Oranga Gisborne New Zealand; 5 Center for Research on Women and Gender College of Medicine University of Illinois Chicago, IL United States; 6 Pathology Department Otago University Dunedin New Zealand; 7 Department of Obstetrics &Gynaecology Christchurch Medical School Otago University Christchurch New Zealand; 8 Department of Biochemistry and Pharmacology The University of Melbourne Australian Centre for the Prevention of Cervical Cancer Melbourne Australia; 9 Australian Centre for the Prevention of Cervical Cancer Melbourne Australia

**Keywords:** uterine cervical neoplasms, cervical intraepithelial neoplasia, early detection of cancer, papillomavirus infections, New Zealand, self-testing, primary health care, Indigenous people, Māori, point-of-care systems, colposcopy, health equity

## Abstract

**Background:**

Māori are the Indigenous people of Aotearoa (New Zealand). Despite global acceptance that cervical cancer is almost entirely preventable through vaccination and screening, *wāhine* Māori (Māori women) are more likely to have cervical cancer and 2.5 times more likely to die from it than non-Māori women. Rural Māori residents diagnosed with cervical cancer have worse outcomes than urban residents. Living in rural Aotearoa means experiencing barriers to appropriate and timely health care, resulting from distance, the lack of community resourcing, and low prioritization of rural needs by the health system and government. These barriers are compounded by the current screening processes and referral pathways that create delays at each step. Screening for high-risk human papillomavirus (hrHPV) and point-of-care (POC) testing are scientific advances used globally to prevent cervical cancer.

**Objective:**

This study aims to compare acceptability, feasibility, timeliness, referral to, and attendance for colposcopy following hrHPV detection between a community-controlled pathway and standard care.

**Methods:**

This is a cluster randomized crossover trial, with 2 primary care practices (study sites) as clusters. Each site was randomized to implement either pathway 1 or 2, with crossover occurring at 15 months. Pathway 1 (community-controlled pathway) comprises HPV self-testing, 1-hour POC results, face-to-face information, support, and immediate referral to colposcopy for women with a positive test result. Pathway 2 (standard care) comprises HPV self-testing, laboratory analysis, usual results giving, information, support, and standard referral pathways for women with a positive test result. The primary outcome is the proportion of women with hrHPV-positive results having a colposcopy within 20 working days of the HPV test (national performance indicator). Qualitative research will analyze successes and challenges of both pathways from the perspectives of governance groups, clinical staff, women, and their family. This information will directly inform the new National Cervical Screening Program.

**Results:**

In the first 15-month period, 743 eligible HPV self-tests were performed: 370 in pathway 1 with POC testing and 373 in pathway 2 with laboratory testing. The positivity rate for hrHPV was 7.3% (54/743). Data collection for the second period, qualitative interviews, and analyses are ongoing.

**Conclusions:**

This Māori-centered study combines quantitative and qualitative research to compare 2 clinical pathways from detection of hrHPV to colposcopy. This protocol draws on rural community practices strengths, successfully engaging Māori from a *whānau ora* (family wellness) approach including *kanohi ki te kanohi* (face-to-face), *kaiāwhina* (nonclinical community health workers), and multiple venues for interventions. It will inform the theory and practice of rural models of the use of innovative technology, addressing Māori cervical cancer inequities and facilitating Māori wellness. The findings are anticipated to be applicable to other Indigenous and rural people in high-income countries.

**Trial Registration:**

Australian New Zealand Clinical Trials Registry (ANZCTR) ACTRN12621000553875; https://anzctr.org.au/Trial/Registration/TrialReview.aspx?ACTRN=12621000553875

**International Registered Report Identifier (IRRID):**

DERR1-10.2196/51643

## Introduction

### Background

Māori are the Indigenous people of Aotearoa (New Zealand). *He Tapu Te Whare Tangata* reflects the veneration of *wāhine* Māori (Māori women) and people with cervices as sacred *whare tangata* (houses of humanity) from a Māori worldview [1]. The overall name of this project “He Tapu Te Whare Tangata—a model for empowering rural solutions” affirms the importance of women’s right to best health.

*Whānau* (families) living in rural Aotearoa face barriers to appropriate and timely health care. This is a complex issue involving distance from main centers, the lack of community resourcing, and low prioritization of rural needs by the national health care system and government. Residents of rural areas have poorer health outcomes than those living in urban areas, and for Māori, who experience inequities in access to care, social determinants of health, and quality of care, this is accentuated [2-4]. To address these barriers, effective solutions for rural Aotearoa should involve both innovative technologies and community empowerment. Using the cervical cancer screening pathway, this partnership project pairs innovative technology with community control of the care pathway to reduce barriers to timely care.

Cervical cancer is one of the few cancers preventable through both vaccination and screening. Screening can detect precancer of the cervix that can be treated, halting the development of a potentially fatal outcome. *Wāhine* Māori are twice as likely to have cervical cancer and 2.5 times more likely to die from it than non-Māori women [5]. Māori living in rural areas diagnosed with cervical cancer have worse outcomes compared with urban residents [6]. These cancers disproportionately affect young *wāhine* Māori and are the second leading cause of cancer death for *wāhine* Māori aged 25-44 years [7,8]. These inequities are unacceptable and are caused by barriers to screening, diagnosis, and treatment [9].

Most cervical cancers occur in women who have not undergone screening or are screened less frequently [10]. The National Cervical Screening Program (NCSP) shows that the target level of 80% of *wāhine* Māori screened within the previous 3 years is not achieved in any region; once screened, there is a failure to achieve a timely diagnosis and treatment of abnormalities for *wāhine* Māori [9]. Fewer *wāhine* Māori have a colposcopy within an appropriate time frame following a high-grade abnormality when compared with non-Māori women. Furthermore, the proportion of women who had no record of any subsequent follow-up at 90 days is 4.4% for European women but 8.6% for *wāhine* Māori [11].

The current national screening approach for cervical cytology involves an invasive procedure using a speculum, which requires attending a clinic with associated transport and other costs. Cervical screening in rural Aotearoa is performed by the primary care nurse or physician, and the tests are sent to a centralized laboratory for analysis. The results are returned to the primary care clinician, who then notifies the patient. If the result is abnormal, the clinician writes a referral letter to a gynecologist requesting an appointment for a colposcopy. Hospital departments then control all further investigations and treatments. Delays can occur at each point, which in turn create barriers to access, timely diagnosis, and treatment [12].

Human papillomavirus (HPV) is the causative agent of cervical cancer. Infection with certain oncogenic (cancer forming) high-risk types of HPV cause changes in the cells of the cervix that can lead to cancer. Compared with cytology, HPV-based screening is a better test; it provides 60% to 70% greater protection against developing invasive cervical cancers [13,14]. The World Health Organization recommends that all countries transition to HPV testing as the primary method for cervical screening [15-17]. It is also internationally recognized that a self-taken swab that uses molecular amplification testing platforms such as polymerase chain reaction is as effective in detecting high-risk HPV (hrHPV) as a clinician-taken sample [18,19].

The present cytology-based NCSP is changing to HPV primary testing this year [20]. HPV self-testing is an equitable tool for cervical screening. Increasing coverage with HPV self-testing will potentially contribute to a more equitable NCSP [20]. A recent randomized controlled trial found that offering an HPV self-test was almost 3 times more likely to result in a cervical screen for underscreened *wāhine* Māori compared with offer of conventional cervical cytology [21]. Qualitative research has shown that HPV self-testing is highly acceptable to *wāhine* Māori, although many who had a positive result felt that more information and support were needed to guide them through the colposcopy process [1,22].

Point-of-care (POC) technology enables on-site HPV testing of self-collected vaginal swabs. The Xpert HPV test by Cepheid has been clinically validated to detect infections with 14 hrHPV types (16, 18, 31, 33, 35, 39, 45, 51, 52, 56, 58, 59, 66, and 68) that are likely to be associated with cervical disease (eg, cervical intraepithelial neoplasia 2+). The Xpert HPV test differentiates between HPV16 and HPV18/45, which are linked to approximately 70% of cervical cancers. The 14 hrHPV types assessed by the Xpert HPV test are associated with >90% of cervical cancers, as demonstrated in a recent Australian study [23]. The sensitivity and specificity of the Xpert HPV test are comparable with those of other well-established HPV central laboratory-based assays and fulfill the World Health Organization’s criteria for use [19,24].

The research center Te Tātai Hauora O Hine (The National Centre for Women’s Health Research Aotearoa, Victoria University of Wellington) was invited to partner with the Iwi (tribal) group Ngāti Pāhauwera Development Trust (NPDT) and Māori health provider Ngāti Porou Oranga (NPO, previously Ngāti Porou Hauora) to try a new clinical pathway for colposcopy using HPV self-taken vaginal swabs, POC testing, and direct referral to secondary services. In the early stages, Tipu Whaipua, a Wairoa-based community steering group formed through a prior research project with NPDT, helped inform the development of the design [25], which was guided by the principles of Ngāti Pāhauwera—*Pakatō i te ata, Pakatō i te ahiahi, Maure mahi Māuri ora* (planning for the future health and well-being of the people) and *Mahia nga māhi o Kahukura* (imagining and creating a better future).

NPO is a Māori primary health organization; it is the sole provider for a challenging geographic area, with a flourishing research and innovation program. The vision of the late Dr Paratene Ngata was “for Ngāti Porou Hauora to lead our own research, as a tikanga (traditional, customary way) and research-based centre of excellence for Hauora Māori” [26]. These principles provided the foundation for this research partnership and the resulting study, “He Tapu Te Whare Tangata—a model for empowering rural solutions.”

This study combines a cluster crossover intervention trial with qualitative work to address inequities in the clinical care pathway to colposcopy for *wāhine* Māori in rural Aotearoa. This study pairs HPV self-testing with POC technology in a novel community-controlled cervical cancer prevention pathway. This easy-to-use technology with a 1-hour turnaround provides quick results for women *kanohi ki te kanohi* (face-to-face) and the ability to provide immediate woman-centered care, including information, *manaakitanga* (support), *whānau* (family) involvement, and shared decision-making. This, in turn, allows for immediate referral to colposcopy appointments for follow-up of positive tests.

### Objectives

The objectives of this study are as follows: (1) to compare 2 pathways of care on the timeliness, referral to, and attendance for colposcopy following hrHPV detection (quantitative) and (2) to assess the acceptability and feasibility of the 2 cervical cancer prevention pathways (qualitative).

## Methods

### Overview

**Figure 1:**
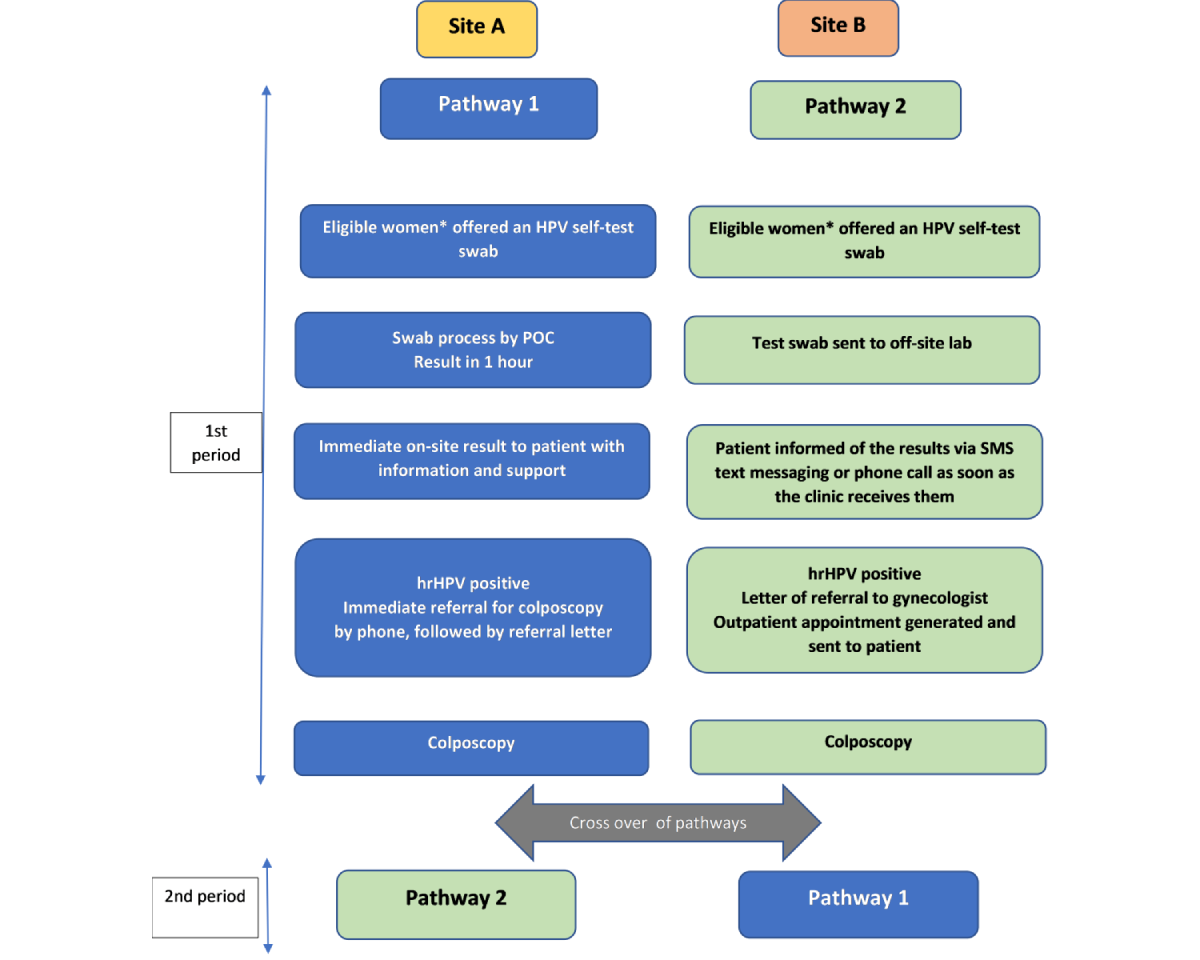
He Tapu Te Whare Tangata—a model for empowering rural solutions: study design. HPV: human papillomavirus; hrHPV: high-risk human papillomavirus; POC: point-of-care. *Eligible women: women aged 24.5 to 69.9 years due or overdue for cervical cytology.

This is a cluster randomized crossover trial, with 2 primary care health practices (study sites) as clusters. Each site was randomized to implement either pathway 1 or pathway 2 in the first study period (15 months) and then crossing over to implement the other pathway in the second study period (15 months). The trial design is summarized in Figure 1.

Pathway 1 is HPV self-testing and POC results within 1 hour, with face-to-face information, support, and immediate referral to colposcopy for women with a positive test result for hrHPV—the community-controlled pathway.

Pathway 2 is HPV self-testing and laboratory analysis, followed by usual results giving, information and support, and standard referral pathways for women with a positive test result for hrHPV—the current standard pathway.

We hypothesize that pathway 1 will improve timely access to colposcopy compared with pathway 2.

We hypothesize that community control of the care pathways is acceptable and feasible for rural Māori communities.

This study is informed by the Kaupapa Māori inquiry paradigm. This paradigm sees being Māori as normal and challenges how Māori have been, and continue to be, constructed within a colonized worldview. It promotes a structural analysis of inequities for Māori, seeking to more fully understand people’s lives and the systemic determinants of their health and wellness [27,28]. In this paradigm, Māori worldviews, ways of knowing, and *mātauranga* Māori (Māori knowledge) are seen as valid and legitimate.

Non-Māori colleagues working collaboratively in a strengths-based approach are included in the team, which is Māori led [29]. With this Māori-centered approach led by *Kaumātua* (Māori elders and knowledge holders), we seek to combine multiple ways of knowing and seeing the world. Māori are involved at all levels, including protocol development, instigation, follow-up, and trialing of this innovative clinical care pathway [25]. Research practices reflect *tikanga* Māori (Māori customs), including the importance of place, relationships, and Māori self-determination [26,27].

The 2 rural North Island community primary health care practices are in Hawke’s Bay and Tairāwhiti on the East Coast of the North Island of Aotearoa at sites A and B, respectively. These 2 primary health care providers serve a predominantly rural and Māori population. The Hawke’s Bay practice is based in 1 health center in a rural town covering an area of approximately 4000 km^2^. The Tairāwhiti practice includes 5 clinics situated in small rural communities, covering a coastline of >200 km.

### Outcomes

The primary outcome is the proportion of women having a colposcopy within 20 working days from the test, for those women with a positive test result for hrHPV.

Secondary outcomes are of those with a positive hrHPV test result:

- Proportion of women having a colposcopy within 40 working days of the test
- Proportion of women having a colposcopy within 20 working days of referral
- Proportion of women having a colposcopy within 40 working days of referral
- Time from test to colposcopy
- Time from referral to colposcopy
- Number of high-grade lesions (cervical intraepithelial neoplasia and carcinoma)
- Planned treatment for women with high-grade histology at colposcopy.

Secondary outcomes include of all women taking the HPV test:

- The proportion of women who test positive for hrHPV
- The proportion of women who test negative for hrHPV
- The proportion of women who have invalid results
- The proportions due for screening, underscreened, or never screened.

Qualitative outcomes are as follows:

- To explore and understand the successes and challenges of both pathways from the perspectives of the governance groups, clinical staff (nurses, doctors, *kaiāwhina* [nonclinical community health workers], and specialists), and women (including *whanau* or family supporters) at both sites
- To understand this new screening pathway to advise and inform a more culturally responsive NCSP.

On the basis of the percentage reported for *wāhine* Māori throughout Aotearoa, we estimated that 56.4% of women in the control arm will have a colposcopy within 20 working days following referral for a positive HPV test [30]. We aim to detect a 50% increase (relative risk 1.5) from 56.4% to 84% in the primary outcome for the intervention arm. A sample size of 80 women (n=40, 50% in each arm) in a 2-period, 2-cluster randomized crossover trial will provide 80% power at 2-sided α of .05 to detect a 50% increase in the intervention arm. With only 2 practices serving as the clusters, the sample size does not account for the cluster effect, which will be considered as a fixed effect in the final analysis.

The order of treatment for each practice has been randomly allocated by the study statistician using computer-generated random numbers. Using the eligible population list for each practice, the research team randomly allocated half of the under- or never-screened women to priority recruitment in the first study period and half to the second study period. This was done to reduce the potential bias from unequal recruitment of under- or never-screened women between the 2 study periods. The lists for priority recruitment were provided to the practices.

As this is a 2-period, 2-cluster randomized crossover trial, each practice acts as a comparator for the other practice during the trial period.

Blinding of participants and clinicians is not possible. The 2 trial statisticians will be unblinded and compile the data set and conduct the statistical analysis.

The inclusion criteria were women aged 24.5-69.9 years eligible for cervical screening in these 2 rural areas. In line with the current NCSP protocol, women can present for the first screen up to 6 months before their 25th birthday and be included. Women can present for screening up to 6 months before their due date and be included [31]. Women who are due for screening or who are underscreened (underscreened is defined as never screened or ≥4 years since last screening) are eligible.

Women with heavy bleeding at the time or those using a vaginal cream that might interfere with the test are asked to return at a later date. Women who opt for cervical cytology or whose general practitioner advises a speculum examination and cervical cytology can also participate in the study by taking an HPV self-taken (or clinician taken) swab first. Women who have symptoms such as abnormal bleeding can still be offered an HPV test, but they will also be seen and assessed by a general practitioner and may require assessment by secondary gynecology services despite a negative hrHPV test.

Primary care clinicians (physicians and nurses) and *kaiāwhina* at both sites received training from the research team on how to obtain informed consent from women. Training was conducted in person or via a video link. Trained clinicians or *kaiāwhina* obtain informed consent from women in both pathways for a copy of their HPV results (and if colposcopy is required, copies of histology results) to be sent to the researchers and the ordering health practitioner.

Women are also asked if they can be contacted later by a qualitative researcher for an interview on their experiences of the pathways (pathway 1 or 2, the HPV self-test, and overall process) and any subsequent follow-up. Other key stakeholders from general practice (management and staff) and the wider community have been identified and invited to participate in an interview.

Physicians, nurses, and *kaiāwhina* at both sites received an HPV update and information sharing session with the research team before recruitment started. These updates included the role of HPV in cervical cancer, the HPV self-test, the role of POC testing, how to obtain informed consent, how to instruct women on taking a self-taken swab, how to follow-up results, the role of the research team, and how the study will run. Training is augmented by regular ongoing contact between the research team, clinic staff, and managers.

All eligible women in both pathways are offered an HPV self-test. The participants receive verbal, written, and pictorial instructions from a physician, nurse, or *kaiāwhina* on how to self-collect a vaginal sample using a nylon-flocked swab (Copán FLOQswabs). Most sample collection takes place at the primary care practice, but some women self-collect the sample in their homes during a *kaiāwhina* visit or take one home from the clinic. Women in pathway 1 are provided information about POC testing. Women in both pathways who have positive HPV results are offered usual support to attend colposcopy. This includes petrol vouchers, transportation, and a support person to accompany them.

### Intervention Description

#### Overview

The site randomized to pathway 1 for the first period nominated ≥2 primary care clinicians and *kaiāwhina* to be trained by qualified members of the research team in the operation and daily quality audit of the Cepheid GeneXpert IV instrument. POC training was overseen by the research team’s molecular scientist in the week before recruitment began. This consisted of orientation and familiarization runs using contrived samples and official training directly from Cepheid via a video link. The technology is robust, but user training and support are vital. Only those holding a training certificate issued by Cepheid are authorized to run patient samples. Regular in-person, on-site POC updates run throughout the trial. User and instrument proficiency testing is built into the implementation process and conforms to the New Zealand Best Practice Guidelines for Point-of-Care Testing [32]. In addition, the research team and site clinicians involved in POC testing are participants in the Royal College of Pathologists of Australasia Quality Assurance Program [33]. The Cepheid GeneXpert IV instruments used in this study are loaned from the Australian Centre for the Prevention of Cervical Cancer.

A 2- to 4-week washout took place between the 2 study periods. The second site received the same training schedule on how to use the Cepheid GeneXpert IV instrument, and the instruments were transported, set up, and tested at the second study site.

#### Pathway 1

Following self-collection, the vaginal sample is processed onsite by the Cepheid GeneXpert IV instrument, taking approximately 1 hour, and the result is documented in the patient management system (PMS). The intention is for women to receive the HPV test result in person onsite, as soon as the test result is available.

Women with hrHPV-positive results receive immediate on-site information about the result, its significance, the next steps, and support. An appointment for colposcopy is made straight away by a phone call to the appointment clerk at the hospital. This phone call is followed up with a referral letter from the referring primary care clinician.

Women with hrHPV-negative results are informed of the result and its significance and advised that they will not need screening for 5 years.

If a woman is unable to stay for an hour to receive her test result, her contact details are taken, and she is contacted as soon as the result is available.

#### Pathway 2

Following vaginal sample self-collection, the woman is asked how she would prefer to receive the result (by SMS text messaging, phone, or in person). The sample is then transported at room temperature to the diagnostic laboratory as part of the practice’s routine laboratory collection. hrHPV genotyping is performed using the Roche Cobas 4800 system. This system detects 14 hrHPV types (16, 18, 31, 33, 35, 39, 45, 51, 52, 56, 58, 59, 66, and 68) and provides a result which distinguishes types 16 and 18 from “other” high-risk types [34]. The use of dry vaginal swabs has been validated in international published studies, and the laboratory is undertaking this testing “off-label” [35,36]. The samples are disposed of in line with the standard pathology laboratory procedures. Test results are sent electronically (encrypted as other routine results) to the ordering primary care clinician and entered into the PMS, as cytology results would be.

Women with hrHPV-positive results are informed of the result and its significance by phone or in person. Information regarding the next steps and support are provided. The primary care clinician sends a referral letter to the colposcopy service at the hospital. The woman is triaged for a colposcopy appointment, and the appointment time is sent to the woman by mail, with or without phone follow-up.

Women with hrHPV-negative results are informed of the result and its significance and advised that they will not need screening for 5 years.

### Data Collection and Analysis

Regular 3 monthly visits to the research sites are made by the members of the research team. These visits include updating the clinic staff on any amendments and audits of the PMS to ensure that the results are documented accurately and coded, given to women, and followed up appropriately. The research team’s molecular scientist visits regularly to audit the POC instruments and sample testing process compliance. The practices have ownership of their data and all the results. Researchers are readily available via phone and email to support practices.

The ordering clinician and practices have the primary responsibility for follow-up. For clinical safety, the unblinded study statistician checks for colposcopy referrals and attendance. This is checked no sooner than 3 months from the hrHPV test date, so the primary outcome is not influenced but ensures that practices can contact any woman who has not yet attended a colposcopy appointment.

Follow-up colposcopy and treatment performed within the public health service are free for Aotearoa citizens and permanent residents. The new NCSP will start in September 2023, and the women in this trial will be registered with the new program appropriately.

Data collection and management are conducted as follows: copies of consent forms, POC test results, and laboratory results are provided to the research team by the study sites and used to track recruitment and monitor safety. Every 6 months, the colposcopy services provide data to the study statisticians: colposcopy attendance and date, colposcopy assessment and outcome, cytology result (if any) at colposcopy, histology, treatment plan, and any treatment provided. No other research team members have access to colposcopy data.

Each person using a health or disability support service in Aotearoa has a unique National Health Index (NHI) number [37]. The research team provides the NHI numbers of the participating women to the National Screening Unit to obtain cervical screening and clinical history at the start of the study period from the NCSP. The NHI number is also used to obtain ethnicity, the deprivation index (socioeconomic status), and age from the NHI data set.

The NHI number is replaced with a unique study code in all research data sets for analysis. No individuals will be identifiable in the reporting of study findings.

Study-specific source documents are maintained in locked file cabinets in locked rooms of Te Tātai Hauora o Hine, Victoria University, Wellington. Electronic data are stored on the secure Victoria University of Wellington server in restricted access folders and password-protected files. The data will be retained for 10 years and then destroyed securely.

Qualitative data collection is conducted as follows: up to 40 women who screen and test positive for HPV 16/18/“Other” (across both study sites and both pathways) and who indicate on their consent form that they are happy to be contacted for an interview will be approached by a *wāhine* Māori researcher. They are invited to take part in an interview at a time and location that suits them and are encouraged to bring any *whānau* (family) or other support. During the interview, they are provided a *koha* (gift) as a small token of appreciation for sharing their experiences and perspectives. Key informants from primary care (eg, clinical staff, nonclinical staff, and management), colposcopy services (eg, clinical and nonclinical staff, and management), and the wider community are invited to be interviewed, either individually or in small groups. Most interviews are conducted in person, but some may be conducted by telephone or Zoom (Zoom Video Communications) depending on COVID-19 precautions and participant preferences [37]. Informed consent is obtained before the interview commences. The researcher takes time to follow culturally appropriate rituals of encounter that help to create a comfortable and safe environment for the interview [38]. Interviews are semistructured around 4 discussion points that aim to elicit rich, nuanced qualitative data. Participants are asked about the acceptability of the screening pathway, perceived level of empowerment and decision-making in the pathway, preferences for screening services, and recommendations for future screening programs. Participants are encouraged to add or ask anything else at the end of the interview. The interviews are audio recorded and transcribed verbatim.

All statistical analyses will be performed using SAS (version 9.4; SAS Institute) and R (version 3.4.2; R Foundation for Statistical Computing). Analyses will be intention to treat and conducted at the individual participant level. All tests of significance are 2-tailed and at a 5% significance level throughout the analyses. Continuous variables will be compared with *t* tests or Mann-Whitney *U* tests, and categorical data will be compared using chi-square tests, as appropriate.

A generalized linear regression model with a binomial distribution will be used to analyze binary outcomes, a linear regression model will be used to analyze continuous outcomes, and the cluster design will be taken into account in the analyses. For the primary outcome, both unadjusted and adjusted analyses for potential covariates measured at baseline will be conducted (eg, deprivation index and age). Unadjusted and adjusted odds ratios will be reported with corresponding 95% CIs and associated *P* values. Relative risks will also be estimated using Poisson regression models with robust error estimates. For the primary outcome, participants with missing data will be assumed as not having a colposcopy within 20 working days. Sensitivity analyses will be conducted for the primary outcome to assess the robustness of the results, which will include per-protocol analyses (where participants with any protocol deviations will be excluded). Additional sensitivity analyses may be considered, such as the potential impact of COVID-19 restrictions or extreme weather events. Subgroup analyses will be conducted for ethnicity, screening history, HPV type, and symptoms (if the numbers are sufficient). Time to notification and time to colposcopy will be analyzed using Kaplan-Meier curves, log-rank tests, and Cox proportional hazard models.

Qualitative data will be analyzed thematically. This will involve reading and rereading the transcripts, coding sections of data inductively, and combining and rearranging the codes to create themes and subthemes [39]. NVivo12 (QSR International) qualitative data analysis software will be used to assist in the coding and theming of data. In reporting the themes, feedback from participants will be written about in a way that groups their talk about common issues and how these issues are interrelated.

Importantly, the governance of this project rests with NPDT and NPO, giving community control of leading-edge POC technology. The study coordinating center is National Centre for Women’s Health Research Aotearoa and includes the roles of the principal investigator, project manager, investigators, research assistants, and Kaumātua. The study coordinating center is responsible for the day-to-day conduct of the trial and supports the practices to participate. This study is a multi-institutional, multidisciplinary, and international research collaboration. There are regular meetings once a week with the research team and once a month with the participating clinics. An external, independent data monitoring committee appointed by the Health Research Council of New Zealand was set up at the request of the research team and meets every 6 months to monitor safety and review recruitment progress and data completion. The National Centre for Women’s Health Research Aotearoa regularly meets the Ministry of Health National Screening Unit to update on the study progress and inform the new NCSP.

Harm to the participants in this trial is very unlikely. In that event, women are eligible to apply for compensation from the national Accident Compensation Corporation, just as if they were injured in an accident, at work, or at home. This study uses the national Health and Disability Ethics Committee’s (HDEC) definitions for serious adverse events, protocol deviations, and protocol violations. Events meeting these definitions are reported to the data monitoring committee and HDEC, along with the actions taken in response to any event.

### Ethical Considerations

Ethics approval was granted on December 2, 2020, by the HDEC (Ref 20/NTB/311). All major protocol amendments are submitted to the HDEC for approval. Locality assessment authorization was granted by the Tairāwhiti District Health Board and Hawke’s Bay District Health Board. Written informed consent is obtained from all participants.

## Results

The first 7 months of the project was spent preparing for the trial (Table 1). Recruitment began in February 2021, and period 1 was completed on April 29, 2022. After a 3-week washout period, period 2 recruitment began on May 23, 2022. This protocol is version 3, dated February 2022. In the first period of 15 months, a total of 743 eligible HPV self-tests were conducted: 370 in pathway 1 with POC testing and 373 in pathway 2 with laboratory testing. The positivity rate for hrHPV was 7.3% (54/743) overall. This is within the globally recognized range of 6% to 15% [40]. The data collection for the second period is ongoing. Once data collection is completed, analysis will report the results for the primary and secondary outcomes. Qualitative data collection and interviews are near completion, and the analysis of themes is ongoing. We expect that final results and analysis will be published in late 2023.

**Table 1:** He Tapu Te Whare Tangata—a model for empowering rural solutions: study timeline.

Task	July 2020 to February 2021	February 2021 to April 2022	May 2022 to July 2023	September 2023
Ethics applications	✓			
Iwi organization and practices consultation	✓			
Recruitment and training primary care practices	✓			
Randomization of sites to pathway 1 or 2		✓		
Intervention commenced		✓		
Data collection		✓		
Qualitative interviews		✓		
Regular progress meetings and visits		✓		
Crossover of pathways			✓	
Qualitative interviews continue			✓	
Analysis				✓
Write up				✓
Dissemination of findings				✓

## Discussion

### Principal Findings

This study combines a cluster crossover intervention trial with qualitative work to address inequities in the clinical care pathway to colposcopy for *wāhine* Māori in rural Aotearoa. This study pairs HPV self-testing with POC technology in a novel community-controlled cervical cancer prevention pathway. The 2 objectives are to compare the 2 pathways of care on the timeliness, referral to, and attendance for colposcopy following hrHPV detection and assess the acceptability and feasibility of the 2 cervical cancer prevention pathways. This protocol draws on the strengths of rural community practices that are successfully engaging Māori from a *whānau ora* (family wellness) approach.

It is known that POC technology is as good as other polymerase chain reaction platforms for testing hrHPV and that self-taken vaginal specimens are as good as clinically taken cervical specimens using these platforms [18,19,24]. There are studies examining POC HPV testing and clinical pathways in low-income countries, but to our knowledge, there is no literature on POC testing and the pathway to colposcopy for disadvantaged populations in high-income countries [41].

### Strength and Limitations

The strengths of this study include that it is informed by a Kaupapa Māori inquiry paradigm. This study was a collaboration between community, Iwi (tribes), rural primary health care clinicians; secondary care gynecology services; and the research team. Health care workers were trained to use POC technology and supported with regular quality assurance. This will lead to the possibility of testing for and treating other common infections in remote or environmentally isolated regions.

The limitations of this study include the recruitment for this study during COVID-19 disruptions and lockdowns and severe flooding and damage to infrastructure due to Cyclone Gabrielle, which affected both research sites. There was a difference in the research sites, as one site had 1 large rural clinic and the other site had 5 small rural clinics; however, there will still be the ability for the analysis to compare each site with itself on each pathway.

### Conclusions

The findings of this study will inform the theory and practice about rural models of the use of innovative technology, addressing Māori cervical cancer inequities and facilitating Māori wellness. Our goal is to enhance outcomes, which necessitates continuous consultation among our partnership (including researchers, community members, Iwi, and primary care providers) with various groups. Our priority for the dissemination of results will be firstly the community, Māori health organizations, and strategic organizations that have been part of the research process. Maintaining the link between academia and Māori communities is part of our commitment to ongoing consultation and dissemination. We have ongoing regular consultation with the National Screening Unit, with the goal that this community intervention, if successful, will directly inform future changes to the NCSP to prioritize Māori and lead to a program that meets the needs of Māori. It is expected that these impacts and solutions may be appropriate for other Indigenous and rural people in high-income countries.

## Appendix

Multimedia Appendix 1 Peer reviewer #12 report for HRC.

Multimedia Appendix 2 Peer reviewer #49 report for HRC.

Multimedia Appendix 3 Peer reviewer #67 report for HRC.

Multimedia Appendix 4 Peer reviewer #91 for HRC.

## Abbreviations

HDEC: Health and Disability Ethics Committee
HPV: human papillomavirus
hrHPV: high-risk human papillomavirus
NCSP: National Cervical Screening Program
NHI: National Health Index
NPDT: Ngāti Pāhauwera Development Trust
NPO: Ngāti Porou Oranga
PMS: patient management system
POC: point-of-care

## Data Availability

All named investigators will have access to the final trial data set. This research aligns with the Te Mana Raraunga Principles of Data Sovereignty, which strictly govern Māori data use and sharing. Data requests with evidence that the proposed use addresses and upholds these principles may be sent to *He Tapu Te Whare Tangata*, rural empowering data manager [42], for consideration by the project data management and principal investigator.
