# Supplementary material for: A Model for Empowering Rural Solutions for Cervical Cancer Prevention (He Tapu Te Whare Tangata): Protocol for a Cluster Randomized Crossover Trial
Source: JMIR Res Protoc. 2023 Sep 14;12:e51643. doi: 10.2196/51643 (PMC10540018; doi:10.2196/51643)
Supplement: Multimedia Appendix 1 [file resprot_v12i1e51643_app1.pdf]

Applicant peer review report

Reviewer # 12

Proposal details

Title	He Tapu Te Whare Tangata: Empowering Rural Solutions
First named investigator	Professor Beverley Lawton (Victoria University of Wellington)

Rationale for research

This project seems to be advocating for the need for 1) point of care testing and 2) a Kaupapa Māori approach to help improve access for Māori women at risk of cervical cancer. The move to HPV testing is already accepted by the Ministry and self-testing has also been researched and is accepted as a useful adjunct to improving access. The proposal outlines that this initiative is most needed in rural communities although the rationale for this is based on one almost 20 year old analysis of routine data. We do not know if there are genuine differences in outcomes for rural Māori compared with urban Māori which is the rationale for undertaking this study in two small rural communities.

Design and methods

This is a mixed methods study with an RCT and some qualitative work.The RCT is a trial of women aged 25-69 who have never been screened or are 3 years or more since their last screen. There are no data in the application on the size of the potential population in these 2 districts i.e. how many women 25 – 69 are there in the 2 districts? What are the current screening coverage rates? How many women are believed to be unscreened? What proportion of women who do attend screening are expected to require colposcopy? Will the women involved be only Māori wahine? Will only women from Ngāti Pahauwera be included? I am unclear whether this Iwi includes all Ngāti Kahungunu in the Wairoa district and so I am unclear as to the prospective numbers involved. Do the researchers have access to the primary care services in Wairoa as there have been major changes to the provision of general practice in the district with the closure of the hospital practice? The recruitment within Ngāti Porou is also not well described. The proposal also does not make clear how participants will be randomised. The study will need to recruit 320 patients to each arm. Given the lack of clarity as to the potential size of the population to be recruited, the lack of detail about key criteria such as past screening, eligibility etc and the lack of details as to how patients will be recruited or randomised, and lack of detail of the expected number requiring colposcopy. The proposed outcome is receiving colposcopy within 20 days? What proportion of women with a positive HPV test currently referred for colposcopy do not attend. Is the 55% quoted the percentage of all those invited or of those that do attend? Is 20 days clinically more important than 6 weeks? Surely it is the DNA rate that is the most important? Overall I am concerned whether this RCT is feasible.

The qualitative arm seems to be recruiting 40 women through hui run in both centres and using semi-structured interviews. Again more detail as to the criteria for the inclusion of women to be interviewed would be helpful. E.g. will the sample include women who have had colposcopy?

Research impact

The argument is that this research has the potential to change the governance of pathways of care in the community. There is little discussion as to the governance of the current pathways or the current involvement of Māori providers in supporting cervical screening/HPV testing. I am not sure how the RCT proposed would provide an argument for changing the governance of the proposed HPV program? It may suggest if successful the benefit of point of care testing of HPV swabs over laboratory testing as suggested. The dissemination plan suggest the team will be able to influence the community with their findings and I am sure they will be capable of helping develop the Māori academic workforce

Research team

Professor Lawton already has a program grant and is running a project grant on HPV testing. She has a well-established track record of research with 62 publications. . Dr Cram is an experienced qualitative researcher and will provide appropriate guidance and support for Anna Adcock who is a doctoral student. Professor Stacie Geller is an overseas expert with a good track record. The rest of the team receiving funding are a mix of staff with goodcommunity links and expertise in providing community based services and some junior researchers with a modest track record in publication. .

General comments

This proposal builds on the current research being conducted by Professor Lawton. The rationale was not entirely clear and the lack of detail with regards the RCT makes this proposal difficult to support. A more detailed and focussed proposal could be worthy of funding.
