# Supplementary material for: A Model for Empowering Rural Solutions for Cervical Cancer Prevention (He Tapu Te Whare Tangata): Protocol for a Cluster Randomized Crossover Trial
Source: JMIR Res Protoc. 2023 Sep 14;12:e51643. doi: 10.2196/51643 (PMC10540018; doi:10.2196/51643)
Supplement: Multimedia Appendix 2 [file resprot_v12i1e51643_app2.pdf]

Applicant peer review report

Health Research Council of New Zealand
Te Kaunihera Rangahau Hauora o Aotearoa

Reviewer # 49

Proposal details

Title	He Tapu Te Whare Tangata: Empowering Rural Solutions
First named investigator	Professor Beverley Lawton (Victoria University of Wellington)

Rationale for research

The rationale for undertaking this research is very sound. Significant inequities exist in cervical screening coverage rates for Māori and this research proposes a change in the way in which cervical screening is undertaken to address a number of the barriers for cervical screening for Māori. Critical aspects of this research are self swabbing for HPV, point of care testing for HPV results and direct referral for colposcopy. If the research is successful then it is likely that a change in the national approach to cervical screening would occur as a result.

Design and methods

The study consists of both quantitative and qualitative strands. The quantitative strand is the strongest aspect of the proposed study. It uses a cluster control cross over method and is powered to detect a 50% increase in timely colposcopies. Women in both arms of the trial will be offered HPV self testing and in the intervention group the self swab is analysed using a point of care testing machine with a 1h turn around. This aspect of the study, is potentially the most problematic (at least in my mind). The authors state that waiting for 1h is a quick result and enable women to have the results given to them kanohi ki te kanohi. 1h might actually be a long time to wait, especially if there are transport barriers and people are reliant on others for transport into the clinic. I would think that there might be a small number of people who do not wait the 1h for the results and this might need to be accounted for. The other aspect of the POC testing was that I could not find a detailed quality plan. The investigators mentioned a clinical safety committee but did not give any details on ensuring reliability of the POC machine. In my previous experience of using POC testing in a cluster control study we ran into significant issues on the reliability of one batch of cartridges used in the machine, resulting in suspension of the trial for a period of time. I would like to see a strengthening of the quality audit processes used.

The qualitative aspect of the study is OK although the data analysis is simply a generic 'thematic' analysis and is not informed by a theoretically derived model. The proposal states that it is Kaupapa Māori research and this could be emphasised a little more in in the description of the qualitative approach to highlight perhaps the involvement of the research whānau in the data analysis and how a KMR lens will inform the data analysis

The study has excellent partnerships with Māori stakeholders and communities and well thought out dissemination strategies that have national implications. The KMR approach is a big strength.

Research impact

As previously noted this research has the potential to inform a national screening programme on addressing an important inequity and is therefore of significant importance. The research team are likely to contribute to Māori health knowledge and building a highly skilled Māori health research workforce (with two early career Māori researchers being involved in the project). Also, as previously noted, a significant strength of the project is its KM approach and the governance of the project by iwi partners.

Research team

The principal investigator is a well established researcher with significant experience in this field and the ability to complete a piece of research like this. There are a number of very experienced researchers in the team as well as a number of very early researchers with minimal research experience.

General comments
