# Supplementary material for: A Model for Empowering Rural Solutions for Cervical Cancer Prevention (He Tapu Te Whare Tangata): Protocol for a Cluster Randomized Crossover Trial
Source: JMIR Res Protoc. 2023 Sep 14;12:e51643. doi: 10.2196/51643 (PMC10540018; doi:10.2196/51643)
Supplement: Multimedia Appendix 3 [file resprot_v12i1e51643_app3.pdf]

Applicant peer review report

Reviewer # 67

Proposal details

Title	He Tapu Te Whare Tangata: Empowering Rural Solutions
First named investigator	Professor Beverley Lawton (Victoria University of Wellington)

Rationale for research

The research aims to address a significant health issue for Maori wahine and whanau. The proposal provides a clear rationale for the proposed research and will build on the existing evidence base that has identified significant disparities in cervical cancer mortality, access to screening, and treatment pathways between Maori and non-Maori. The proposed research aims to demonstrate the effectiveness of a community and iwi led Kaupapa Maori intervention to improve access to timely screening, diagnosis, and treatment to improve equity of outcomes for Maori.

Design and methods

The proposed research has a comprehensive and robust mixed methods designincorporating an RCT and qualitative research. The proposed study designs are a strength from a clinical perspective and importantly incorporates community and kaupapa Maori approaches including community and iwi control over the research. The research is well designed for Maori and for the communities in which the research is based and will be conducted. The method will address the research aims and can be delivered within the time frames proposed.

The primary outcome is "Proportion of women with an HPV positive test having a colposcopy within 20 working days." Was there consideration among the researchers to follow up over the longer term to identify differences in mortality (as identified by Goal 1 - "ultimately reduce cervical cancer for Māori").

Further consideration is also needed in relation to the power of the study. Your study may be under powered if your change in outcome is less than a 50% increase. Furthermore, the primary outcome may not be achieved if there is not capacity at the colposcopy clinics to complete the colonoscopies within the 20 day time frame.

Research impact

The research findings has the potential to address a significant health issue that has implications for Maori health and equity, healthcare systems and national cervical screening policies, models of care including care pathways, culturally responsive care, and for primary and rural health services. The research could also be an exemplar for how research can be conducted with iwi, Maori providers, and communities.

The researchers have engaged necessary stakeholders (eg DHB and National Screening Unit, providers) which will increase the likelihood of the research translating into policy and changes in care pathways.

Research team

The researchers have assembled a multidisciplinary team with an appropriate mix of clinicians, expertise in women's health and Maori health and primary care, Hauora and iwi, technical support, and importantly researchers with iwi affiliation to the community where the research is being conducted. Members of the team have the necessary research experience, knowledge, and designated FTE to deliver on the proposed research objectives. The lead investigators have a track record of securing previous HRC funding and delivery of outputs.

General comments
