# Supplementary material for: A Model for Empowering Rural Solutions for Cervical Cancer Prevention (He Tapu Te Whare Tangata): Protocol for a Cluster Randomized Crossover Trial
Source: JMIR Res Protoc. 2023 Sep 14;12:e51643. doi: 10.2196/51643 (PMC10540018; doi:10.2196/51643)
Supplement: Multimedia Appendix 4 [file resprot_v12i1e51643_app4.pdf]

Applicant peer review report

Reviewer # 91

Health Research Council of New Zealand
Te Kaunihera Rangahau Hauora o Aotearoa

Proposal details

Title	He Tapu Te Whare Tangata: Empowering Rural Solutions
First named investigator	Professor Beverley Lawton (Victoria University of Wellington)

Rationale for research

The project presents an excellent rationale for the research.It not only addresses a significant health issue for rural Maori women, but builds on their previous and ongoing work aroundHPV self-testing in Maori women. The combination of community control of the care pathway anduse of new technology will enable on site POC resultswith the aim of reducing barriers to timely care for rural Maori women.The only concerns being those outside the control of the team such as the lack of funding by MOH in implementing primary HPV screening, but hopefully this research will add robust evidence to both the MOH and NCSP to push for timely implementation of a primary HPV screening program.

Design and methods

This study is well designed to address the research aims and objectives, and covers all the above objectives clearly.As stated the overall aim is to explore the acceptability and feasibility of community control pathways combined with innovative technology to overcome barriers existing for rural Maoi communities.As notedpreviously theproposal will depend heavily on timely access to treatment and the ability of DHB clinic to process a potential increase in patients in a timely way . There are both workforce and funding issues beyond the control of the team and this potentially may lead to raising patient expectations of timely care and the team no being able to ensure this is achievable.What processes are in place to deal with these issues ?

Research impact

The proposal clearly outlines both the benefits and opportunities for this research to contribute to creation of Maori Knowledge and to influence policy, practice, and rural health services.

Research team

This research team is highly qualified to carry out this work and has the right mix of expertise within the team to achieve the aims and objectives as proposed.They haveestablished networks both within Maori communities and within government to achieve the impacts

as stated within the proposal.

General comments
